# Two-Dimensional Transition Metal Dichalcogenides: Synthesis, Biomedical Applications and Biosafety Evaluation

**DOI:** 10.3389/fbioe.2020.00236

**Published:** 2020-04-07

**Authors:** Xiaofei Zhou, Hainan Sun, Xue Bai

**Affiliations:** ^1^Faculty of Science and Technology, Bohai Campus, Hebei Agricultural University, Cangzhou, China; ^2^Shandong Vocational College of Light Industry, Zibo, China; ^3^School of Public Health, Shandong University, Jinan, China

**Keywords:** 2D TMDCs, synthesis, modification methods, biomedical application, biosafety evaluation

## Abstract

Recently, two-dimensional transition metal dichalcogenides (2D TMDCs) have drawn certain attentions in many fields. The unique and diversified electronic structure and ultrathin sheet structure of 2D TMDCs offer opportunities for moving ahead of other 2D nanomaterials such as graphene and expanding the wide application of inorganic 2D nanomaterials in many fields. For a better understanding of 2D TMDCs, one needs to know methods for their synthesis and modification, as well as their potential applications and possible biological toxicity. Herein, we summarized the recent research progress of 2D TMDCs with particular focus on their biomedical applications and potential health risks. Firstly, two kinds of synthesis methods of 2D TMDCs, top-down and bottom-up, and methods for their surface functionalization are reviewed. Secondly, the applications of 2D TMDCs in the field of biomedicine, including drug loading, photothermal therapy, biological imaging and biosensor were summarized. After that, we presented the existing researches on biosafety evaluation of 2D TMDCs. At last, we discussed major research gap in current researches and challenges and coping strategies in future studies.

## Introduction

**FIGURE 1:**
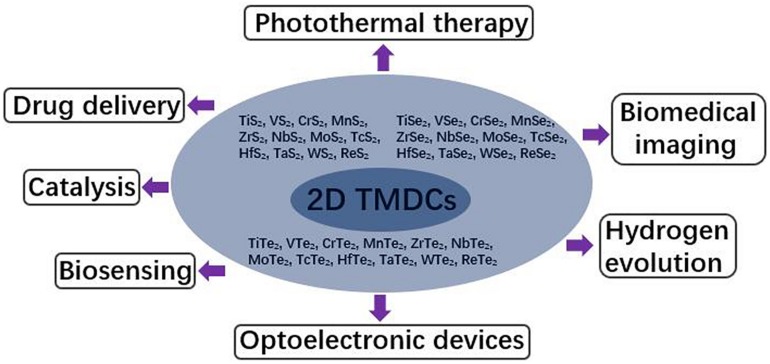
Summary of the types and applications of 2D TMDCs.

As the most well-known two-dimensional (2D) nanomaterials, graphene and graphene derivatives have been receiving great attention due to their fascinating physicochemical properties (Liu et al., 2012b). In recent years, a newly emerging kind of 2D nanomaterial, two-dimensional transition metal dichalcogenides (2D TMDCs) got a lot of attention, whose generalized formula is MX_2_, where M represents transition metal and X represents chalcogen. M comprises of transition metals from the IVB to VIIB group, including Ti, V, Cr, Mn, Zr, Nb, Mo, Tc, Hf, Ta, W, and Re; X represents the chalcogenide elements, sulfur, selenium, and tellurium of the sulfur group (Figure 1). The metal coordination of 2D TMDCs is generally either trigonal prismatic or octahedral (Chhowalla et al., 2013). The synthetic method is gradually perfect and the present synthetic methods could be divided into two categories, top-down and bottom-up methods (Chen et al., 2015; Liu and Liu, 2018). In general, 2D TMDCs synthesized through top-down methods are mainly used in biomedical field, while 2D TMDCs prepared through bottom-up methods are mostly applied photoelectric devices and catalysis field.

The same as graphene and graphene derivatives, 2D TMDCs possess ultrathin structure and high surface-area-to-mass ratio (Chhowalla et al., 2013), which are favorable for loading multiple molecules, such as organic molecules (Liu et al., 2014b; Yin et al., 2014; Ariyasu et al., 2017) and genes (Kim et al., 2016), through van der Waals interaction or covalent bonds. Compared with graphene, the electronic structure of 2D TMDCs exhibit great differences. Graphene is an indirect band gap semiconductor, while the unique electronic structure of 2D TMDCs makes them direct band gap semiconductors (Chhowalla et al., 2013). In addition, only through different chemical modification, graphene can realize the diversity of properties. Two-dimensional TMDCs are a large family, including 36 kinds of materials. The band gap energy of 2D TMDCs varies with the composition of elements, and the properties of 2D TMDCs can be diversified without surface modification (Chhowalla et al., 2013). Thus the unique structure and photoelectric properties of 2D TMDCs make them more widely used than graphene in many fields. In the field of optoelectronics, 2D TMDCs can be used in catalysis, hydrogen evolution and a variety of optoelectronic devices, such as transistors (Zhao et al., 2019), photodetectors (Park et al., 2017), photoelectric modulators (Li et al., 2017), electrodes (Wang J. et al., 2017) and battery diaphragms (Ghazi et al., 2017). In addition, MoS_2_ nanosheets, the typical representative of 2D TMDCs, exhibits thickness-dependent photoelectric effect (Pak et al., 2018) and thickness-dependent photoacoustic signal (Chen et al., 2016). The most noteworthy is that 2D TMDCs can be excellent nanoplatform for biomedical application, such as drug delivery (Liu et al., 2014b; Han et al., 2016; Yang et al., 2018), biosensing (Zhu et al., 2013; Farimani et al., 2014; Yuan et al., 2014). High photothermal/photoacoustic conversion coefficient enables 2D TMDCs promising for photothermal therapy (Jin et al., 2003; Li et al., 2012) and biomedical imaging (Ma et al., 2017; Figure 1).

The broad application prospect of 2D TMDCs greatly increases human exposure opportunities. Two-dimensional TMDCs as airborne particles will reach human respiratory system which are generated during production, usage, transportation and disposal of 2D TMDCs-based products. The applications of 2D TMDCs in biomedical field require their injection into circulation and reaching human organs and tissues. In addition, solar energy is the most easily available and cheapest light energy. The solar disinfection of drinking water mostly depends on the ultraviolet in solar energy, which only accounts for 4% of the solar energy, leading to the low efficiency of solar disinfection. Therefore, it is urgent to develop new materials that can harvest visible light for water disinfection, so as to accelerate the water disinfection effect of solar energy. It has been proved that few-layered MoS_2_ membrane produced ROS and kill bacteria in water through absorbing 50% solar energy (Liu et al., 2016). The application of 2D TMDCs in drinking water disinfection will increase the possibility of human contact with 2D TMDCs through digestive system. Many possible exposure pathways of 2D TMDCs will greatly increase the chance of contact with human beings. Once entering human body, 2D TMDCs will disturb the normal physiological state. The biosafety evaluation of 2D TMDCs is of great significance to human health.

The existing limited studies have shown that cell viability and some other cell behaviors, such as cell proliferation (Zou et al., 2017), oxidative stress (Yang et al., 2014), cell autophagy (Zhou et al., 2019), and metabolism (Yu Y. et al., 2017) were affected by 2D TMDCs. In this review, I will give a brief summary based on present progress on the synthesis and surface modification methods, biomedical applications, and biosafety evaluation of 2D TMDCs. The challenges and prospects of 2D TMDCs in synthesis and biosafety evaluation will also be discussed.

## Synthesis of 2D TMDCs

At present, a variety of preparation methods including mechanical exfoliation (Novoselov et al., 2004; Li et al., 2013), liquid phase exfoliation (Coleman et al., 2011; Vega-Mayoral et al., 2016), chemical exfoliation (Eda et al., 2011; Zeng et al., 2011), chemical vapor deposition (Kim et al., 2012; Liu K.-K. et al., 2012; Wang et al., 2013), and solvothermal synthesis (Peng et al., 2001b; Ramakrishna Matte et al., 2010), have been developed to synthesize 2D TMDCs with single or few layers. These methods can be divided into two categories: top-down (get layered nanomaterials from bulk crystals through different exfoliation ways) and bottom-up approaches (use atoms or molecules as precursors to grow into layered nanomaterials under special conditions) (Fiori et al., 2014).

### Top-Down Synthesis

#### Mechanical Cleavage

Mechanical cleavage is the most typical top-down method. In the mechanical cleavage process, the adhesive force of scotch tape is used to obtain monolayer or few-layer structures from bulk crystals. To date, many kinds of ultrathin 2D TMDCs have been synthesized in virtue of mechanical cleavage method (Novoselov et al., 2004; Splendiani et al., 2010; Radisavljevic et al., 2011). Mechanically exfoliated ultrathin 2D TMDCs are equipped with personal advantages and disadvantages. Ultrathin 2D TMDCs prepared through this method are highly crystalline nanosheets with large size and few defects, which are suitable for electronic devices and fundamental studies of intrinsic physicochemical properties. However, the production rate is low, and the size and thickness are hard to control. The substrate is needed to support the nanosheet. The 2D TMDCs prepared by this method is difficult to meet the needs of biomedicine (Chen et al., 2015).

#### Liquid Exfoliation

**FIGURE 2:**
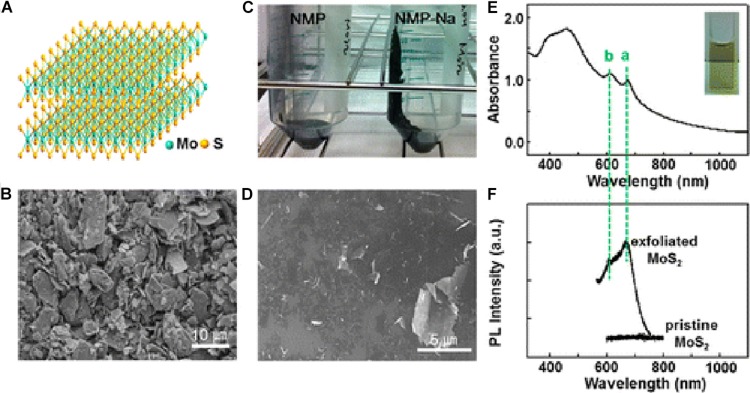
Liquid exfoliation of bulk MoS_2_. **(A)** The structure of MoS_2_, **(B)** SEM images of pristine MoS_2_ powder, **(C)** photo image for the effect of exfoliation with NaOH (the right side) and without NaOH (the left side, the control sample), **(D)** SEM image of the exfoliated MoS_2_ nanosheets with NaOH in NMP, **(E)** UV-vis spectrum (inset: photo image of the MoS_2_ dispersion), and **(F)** photoluminescence spectrum. The “a” and “b” peaks can be assigned to the characteristics of a 2H-MoS_2_ nanosheet and correspond to the smallest direct transition. Reproduced with permission from Bang et al. (2014).

Liquid exfoliation is another typical top-down method. Liquid exfoliation could realize successful exfoliation of bulk crystals via ultrasonication in specific solvent (Figure 2; Dines, 1975; Joensen et al., 1986; Bang et al., 2014; Yong et al., 2014). By sonicating, the weak van der Waals interaction but not strong covalent bonds in-plane could be broken down. Therefore, proper ultrasonic intensity and ultrasonic time are critical to realize the successful exfoliation of bulk crystals. The main function of solvent molecules is to stabilize exfoliated nanosheets and inhibit their reassemble. The solvent molecules with appropriate surface energy bind to the surface of nanosheets via van der Waals interaction. Hence the matching degree of surface free energy between solvent molecules and nanosheets is very important to improve the exfoliation efficiency. At present, the common solvents are mainly organics, such as dimethylformamide (DMF) and N-methyl-pyrrolidone (NMP) (Jawaid et al., 2016). To date, multiple ultrathin 2D TMDCs have been synthesized through liquid exfoliation, such as MoS_2_ (Bang et al., 2014), WS_2_ (Vega-Mayoral et al., 2016), NbSe_2_, TaSe_2_, and NiTe_2_ (Coleman et al., 2011). Liquid exfoliation makes up for some deficiencies of mechanical cleavage, realizing the large-scale preparation of ultrathin 2D TMDCs with good photoelectric properties. However, the organic solvents used in liquid exfoliation process are undesirable in following applications, and it is difficult to produce single-layer 2D TMDCs through this method. Therefore, it is necessary to further improve the experimental conditions for the large-scale synthesis of monolayer 2D TMDCs in non-toxic solvent.

#### Chemical Exfoliation

Chemical exfoliation method is to insert intercalators into the interlayer of the bulk crystals with the help of ultrasonication in water, realizing the successful exfoliation of bulk crystals (Eda et al., 2011; Lukowski et al., 2013). The most common intercalators are organometallic compounds, such as butyl lithium, naphthyl sodium, etc. During the synthesis process, intercalators are firstly intercalated into interlayer of bulk TMDCs in water or ethanol. Then the bulk TMDCs are exfoliated into ultrathin nanosheets under sonication. Now the insertion of intercalator into bulk TMDCs has been realized in battery, and the amount of intercalator was regulated by controlling the voltage (Zeng et al., 2011). Chemical exfoliation has been used to prepare a variety of ultrathin 2D TMDCs without the use of toxic organic solvents in the synthesis process. This method could meet the needs of the biomedical applications of ultrathin 2D TMDCs.

### Bottom-Up Synthesis

#### Chemical Vapor Deposition

**FIGURE 3:**
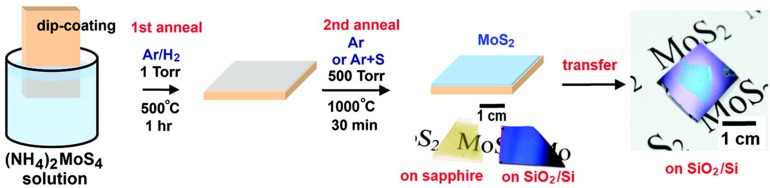
Schematic illustration of the two-step thermolysis process for the synthesis of MoS_2_ thin layers on insulating substrates. The precursor (NH_4_)_2_MoS_4_ was dip-coated on SiO_2_/Si or sapphire substrates followed by the two-step annealing process. The as-grown MoS_2_ film can be transferred onto other arbitrary substrates. Reproduced with permission from Liu K.-K. et al. (2012).

Chemical vapor deposition is a typical bottom-up method. The reaction process is to expose the reaction precursor to the substrate under high temperature and pressure. The role of reaction precursors is to provide transition metal atoms and chalcogenide atoms, respectively, and react to generate ultrathin 2D TMDCs (Figure 3). Finally, the reaction product was deposited on the substrate, thus the ultrathin 2D TMDCs were obtained (Lee et al., 2012; Liu K.-K. et al., 2012; Wang et al., 2013, 2014; Ling et al., 2014). Ultrathin 2D TMDCs nanosheets prepared through this method possess excellent electronic property and high crystal quality. However, high vacuum and high temperature are necessary in the synthesis process. And the use of substrate increases the transfer process of nanosheets.

#### Solvo-Thermal Synthesis

Solvo-thermal synthesis is another bottom-up method. By solvo-thermal method, ultrathin 2D TMDCs could be obtained from precursors under the condition of specific solvent and specific reaction time (Peng et al., 2001a, b; Cao et al., 2014). The results show that after the reaction of molybdic acid or tungstic acid with thiourea at 773K for 3 h, the ultrathin MoS_2_ or WS_2_ nanosheets can be prepared. The strength of this method is that it could realize the high-yield preparation of ultrathin 2D TMDCs at a lower cost. Hence this method will be promising for industrial application of 2D TMDCs. The shortage of solvo-thermal synthesis is that single-layer nanosheet is difficult to be obtained.

### Surface Modification

**FIGURE 4:**
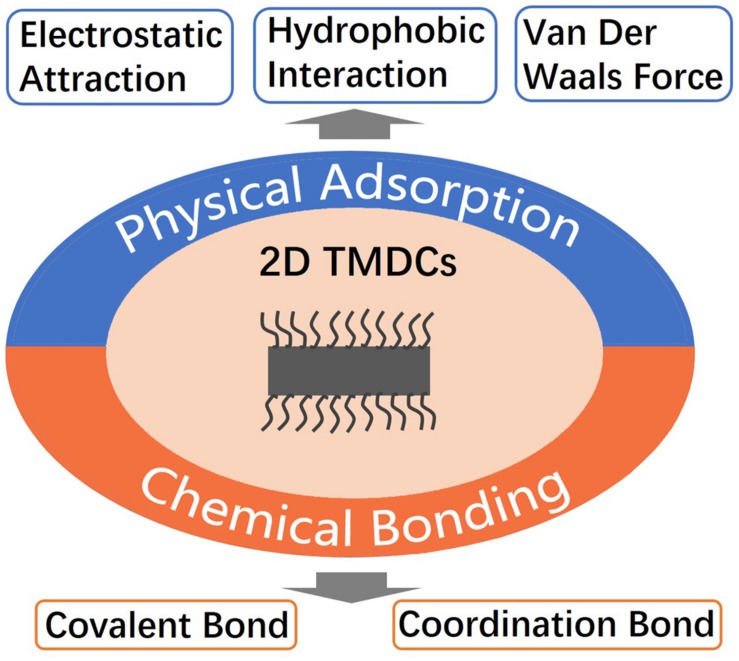
Two kinds of surface modification methods of 2D TMDCs.

**FIGURE 5:**
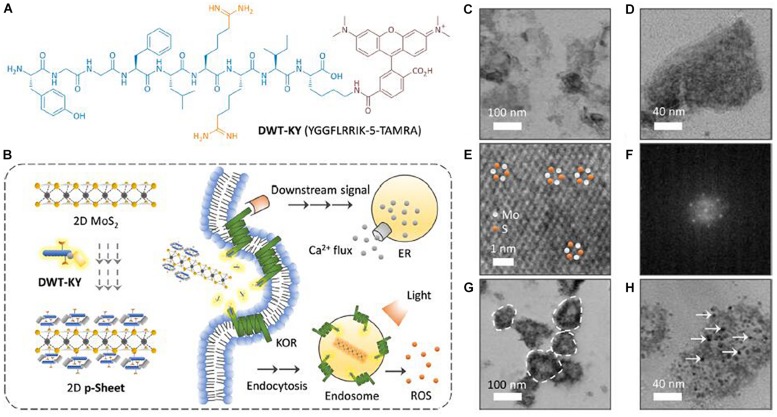
Surface modification of 2D TMDCs through physical adsorption. **(A)** Structure of the fluorescent agonist probe DWT-KY (YGGFLRRIK-5-TAMRA, where TAMRA is 5-carboxu etramethylrhodamine) for KOR binding. **(B)** Schematic illustration of the formation of a 2D p-Sheet between 2D MoS_2_ and DWT-KY and the use of the material ensemble for targeted activation of a KOR. This then leads to (1) activation of a downstream signaling pathway to release Ca^2+^ flux from endoplasmic reticulum and (2) endocytosis of the material that can release ROS intracellularly upon light irradiation. **(C–E)** High-resolution transmission electron microscopy (HRTEM) of 2D MoS_2_. **(F)** Fast Fourier transform pattern of a selected area from HRTEM of 2D MoS_2_. **(G,H)** HRTEM image of 2D p-Sheet (DWT-KY/2D MoS_2_ = 1 μM/35 μg mL^–1^) [the dashed circles in **(G)** highlight several representative 2D p-Sheets, and the arrows in **(H)** highlight several agonist probe particles adhered to the surface of 2D MoS_2_]. Reproduced with permission from Dou et al. (2017).

**FIGURE 6:**
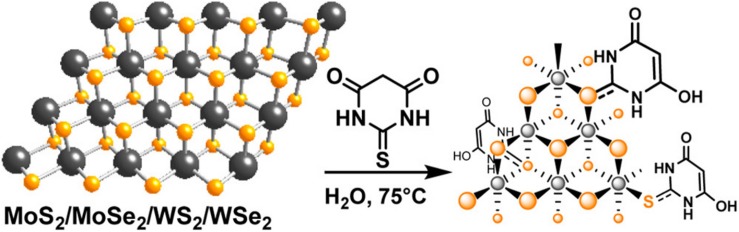
Surface modification of 2D TMDCs through chemical bonding. Synthesis of thiobarbituric acid (TBA)-modified MoS_2_/MoSe_2_/WS_2_/WSe_2_ with high organic molecule coverage. Reproduced with permission from Presolski et al. (2017).

Due to the high surface area-mass-ratio, abundant atoms of 2D TMDCs are exposed to the outside, leading to super high surface free energy (Chhowalla et al., 2013). Hence the ultrathin 2D TMDCs are lack of stability in physiological conditions. To improve the stability, dispersibility and potential application in multiple fields, surface modification of 2D TMDCs are needed urgently. There are two kinds of surface modification methods (Figure 4), including physical adsorption (Figure 5; Yong et al., 2014; Han et al., 2016; Dou et al., 2017) and chemical bonding (Figure 6; Shen et al., 2016; Oudeng et al., 2018). Physical adsorption is mainly through the electrostatic attraction, hydrophobic interaction, and van der Waals force to achieve the surface modification of nanosheets. For example, doxorubicin, as a small molecule drug, could be adsorbed on the surface of MoS_2_ nanosheets through hydrophobic interaction and improved the efficiency of MoS_2_ nanosheets in killing cancer cells (Liu et al., 2014b; Yin et al., 2014). In addition to small molecules, large molecules such as DNAs and proteins can also be connected to the surface of 2D TMDCs through non-covalent interaction. The dye labeled single-strand DNA probe can be adsorbed to the surface of MoS_2_ nanosheets through van der Waals interaction between the bases and the surface of MoS_2_ nanosheets, thus realizing the effective detection of DNA (Han et al., 2016; Lu et al., 2017). After being mixed with WS_2_ solution for 3 h at room temperature, bovine serum album was successfully adsorbed on the surface of WS_2_ nanosheets through van der Waals interaction (Yong et al., 2014).

Chemical bonding achieves surface modification of the nanosheets with the help of covalent bond or coordination bond (Yong et al., 2014; Shen et al., 2016; Han et al., 2017; Oudeng et al., 2018). The chemical bond is much stronger than van der Waals force. At present, the commonly used chemical bonding method is to form a transition metal-sulfur bond between the molecules with sulfur atom at the end and the transition metal atom on the surface of 2D TMDCs, realizing the surface modification of 2D TMDCs. When folic acid-PEG-SH was mixed with MoS_2_ nanosheets for 5 h under ultrasonication, the surface modified MoS_2_ nanosheets were obtained, which could specifically target the cells over expressing folate receptor (Oudeng et al., 2018). In addition to the surface polyethylene glycol (PEG) modification of 2D TMDCs, some small molecules such as thiobarbituric acid (TBA) can also be connected to the surface of MoS_2_ nanosheets through the formation of Mo-S bond. Various surface modifications of MoS_2_ nanosheets can be realized by NH_2_ on TBA (Figure 6; Presolski et al., 2017). In addition, 2D TMDCs could also be functionalized by *in situ* polymerization with polymer (Shen et al., 2016).

The surface modification of 2D TMDCs will widen their application range and stimulate their application potential. For example, multiple functionalized 2D TMDCs have been used in drug delivery, photothermal therapy and tumor imaging. However, the functionalization methods of 2D TMDCs are insufficient. Massive efforts are still needed to complete the surface modification methods of 2D TMDCs.

## Biomedical Applications of 2D TMDCs

In recent years, with the rapid development of preparation methods and surface functionalization methods, 2D TMDCs with various properties are on the crease, which greatly promote their application in biomedical field. Current biomedical application of 2D TMDCs can be divided into four categories: drug delivery, photothermal therapy, biological imaging, and biosensing.

### Drug Delivery

As a drug carrier, 2D TMDCs has three advantages: firstly, compared with liposomes and micelles, 2D TMDCs with stronger stability can achieve sustained release of drugs and avoid explosive drug release; secondly, the super high surface area of 2D TMDC provides a large number of anchor sites for upload molecules; and thirdly, the surface decoration of 2D TMDCs can be achieved easily through physical adsorption or chemical bonding. Ultrathin 2D TMDCs can efficiently upload a variety of drug molecules, including doxorubicin, 7-ethyl-10-hydroxycamptothecin, chitosan, photodynamic reagent, etc (Liu et al., 2014b; Yin et al., 2014; Han et al., 2016; Yang et al., 2018). For example, a functionalized MoS_2_ nanosheets was developed for combined cancer therapy. After MoS_2_ nanosheets synthesis, lipoic acid modified PEG (LA-PEG) was linked to the surface of MoS_2_ nanosheets to improve biocompatibility and physiological stability. The strong NIR absorbance makes the MoS_2_-PEG nanosheets promising candidate for photothermal therapy. Due to the super high surface-area-to-mass ratio, MoS_2_ nanosheets exhibited a high drug loading percent for chemotherapy drugs, such as doxorubicin, photodynamic agent chlorine e6 and 7-ethyl-10-hydroxycamptothecin. *In vitro* cell culture tests and *in vivo* cancer treatment, MoS_2_-PEG with doxorubicin uploaded can be utilized for chemotherapy and combined photothermal (Liu et al., 2014b). As a platform for immunotherapy, MoS_2_–PEG–CpG was also constructed. After MoS_2_ nanosheets were synthesized by chemical exfoliation, the nanosheets were functionalized by cytosine–phosphate–guanine (CpG) and PEG, and finally the MoS_2_–PEG–CpG nanoconjugates were formed. MoS_2_–PEG–CpG remarkably promoted the intracellular accumulation of CpG and stimulated the production of proinflammatory cytokines, elevating the immune response level. Not only that, when co-cultured with macrophage-like cells, MoS_2_–PEG–CpG nanoconjugates effectively reduced the proliferation activity of cancer cells upon NIR irradiation, suggesting a new strategy for cancer treatment (Han et al., 2017). In addition to drug delivery, 2D TMDCs can also be used as an excellent gene delivery platform (Kou et al., 2014; Kim et al., 2016). Polyethylenimine (PEI) and PEG were attached to the surface of MoS_2_ nanosheets via disulfide bonds. Then DNA interacted with the MoS_2_–PEI–PEG hybrid nanocomposite by electrostatic interaction, and a complex with high stability was formed. Upon near infrared light irradiation, photothermally triggered endosomal escape was induced and the polymers were detached from surface of MoS_2_ nanosheets by the intracellular glutathione, resulting in gene release from the hybrid nanocomposite. This sequential process significantly enhanced gene delivery efficiency without severe cytotoxicity. This MoS_2_ nanocomposite provided a controllable platform to deliver genes into cells (Kim et al., 2016). In another system, the amino end of MoS_2_-PEG-PEI nanosheets bound to negatively charged siRNA. As a critical regulator of cell cycle, a well-known oncogene Polo-like kinase 1 was investigated. After the knockdown of Polo-like kinase 1 with siRNA carried by MoS_2_-PEG-PEI nanosheets, the interfering efficiency and transfection effect were measured respect through qPCR, western blot and apoptosis assay. All the results suggested that as a novel nanocarrier, MoS_2_-PEG-PEI nanosheets exhibited high gene-carrying ability, good biocompatibility, as well as reduced cytotoxicity (Kou et al., 2014). Two-dimensional TMDCs have great potential to become excellent gene carrier. In addition, graphene, another 2D nanomaterial, which has been studied more recently, can also be used for drug loading. The drug molecule realized the drug upload through the amide reaction with the carboxyl group on the surface of graphene (Zhang M. et al., 2013, 2017). However, the smooth progress of amide reaction requires the introduction of condensation agent and dehydrating agent, which greatly increases the complexity of this process. Because 2D TMDCs have a sandwich structure, in which the metal atoms are in the middle of the layered structure and the chalcogen atoms are on the surface, the drug molecules can be directly uploaded through the interaction with chalcogen atoms on the surface, and the reaction process is relatively simple (Chhowalla et al., 2013; Liu et al., 2014b). Compared with graphene, 2D TMDCs are more suitable for drug loading.

### Photothermal Therapy

**FIGURE 7:**
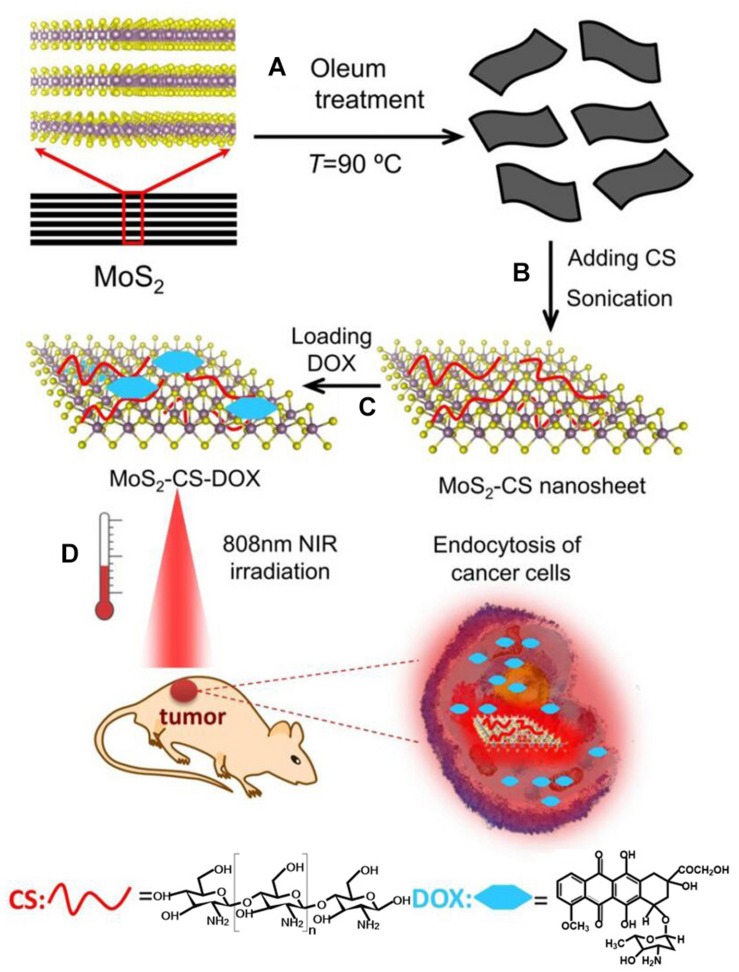
Schematic illustration of MoS_2_-CS nanosheets as a NIR photothermal triggered drug delivery system for efficient cancer therapy. **(A,B)** Oleum treatment exfoliation process to produce single layer MoS_2_ nanosheets and then modified with CS, **(C)** DOX loading process, and **(D)** NIR photothermal-triggered drug delivery of the MoS_2_ nanosheets to the tumor site. Reproduced with permission from Yin et al. (2014).

The principle of photothermal therapy is to use laser to generate heat and induce hyperthermia within tumor tissue, which causes denaturation of proteins, disruption of cell membrane and irreversible damage to cancer cells (Shibu et al., 2013). However, the non-specific high-intensity laser treatment damages both normal and tumor tissues, causing serious side effects (Huang et al., 2007). The introduction of photothermal agents can effectively improve the specificity of laser, which means that it can selectively generate heat within tumor tissue at a relatively low laser intensity, thus reducing the damage to normal tissue (Jin et al., 2003; Li et al., 2012). Because of strong light absorption ability in the near-infrared window area, 2D TMDCs have been used as photothermal agents in photothermal therapy and have played an effective role in removing cancer cells *in vivo* (Figure 7; Yin et al., 2014; Qian et al., 2015; Wang et al., 2015a; Han et al., 2017). The mass extinction coefficient of ultrathin MoS_2_ nanosheets with thickness of about 1.54 nm was 29.2 L g^–1^ cm^–1^, which was 7∼8 folds higher than that of graphene. When the concentration of MoS_2_ was between 38 and 300 ppm, the solution temperature can rapidly raised to 40°C by irradiation with a wave laser at 800 nm. The *in vitro* studies in human cervical cancer cell line showed that almost all cells were killed after incubation with MoS_2_ nanosheets for 20 min under 800 nm near-infrared light (Chou et al., 2013). As another typical representative of 2D TMDCs family, WS_2_ nanosheets, similar to MoS_2_ nanosheets, also has high photothermal conversion coefficient. It was found that the mass extinction coefficient of WS_2_-PEG nanosheets at 808 nm reached 23.8 L g^–1^ cm^–1^, then its photothermal therapy ability was tested by *in vivo* experiments. After WS_2_-PEG nanosheets was intravenously injected into mice, when irradiated with 808 nm laser at the intensity of 0.8 W cm^–2^, the tumor surface temperature of mice reached 65°C within 5 min, and the tumor could be completely removed without obvious recurrence, thus greatly improving the survival rate of mice (Cheng et al., 2014). In addition to 2D TMDCs, 2D graphene and graphene derivatives can also be used in photothermal therapy because of their high light absorption efficiency in the near-infrared window (Liu W. et al., 2019; Roy et al., 2019). Compared with graphene, the mass extinction coefficient of MoS_2_ nanosheets with thickness of 1.54 nm was 7.8 times that of graphene in the near infrared range, which suggested that 2D TMDCs may be a better choice than graphene in photothermal treatment (Chou et al., 2013).

### Biomedical Imaging

Based on the unique chemical composition and the special physical and chemical properties of layered structure, 2D TMDCs can be effectively used in a variety of biological imaging. At present, the application of 2D TMDCs in the field of biological imaging can be divided into three categories: fluorescence labeled imaging, photoacoustic imaging and X-ray computed tomography (CT) imaging. When 2D TMDCs are used for fluorescence labeled imaging, it is necessary to label 2D TMDCs with fluorescent molecules, and then the fluorescent imaging of cells or tissues can be realized by targeting specific cells or tissues with fluorescent labeled nanosheets (Dou et al., 2017; Ma et al., 2017). For example, after the peptide ligand (TAMRA DN1K) linked with the fluorophore group was connected to MoS_2_ nanosheets, the nanosheets can recognize the liver cancer cells or liver cancer tissues with high expression of CD47. At this time, TAMRA DN1K on MoS_2_ nanosheets combined with CD47 on cell surface, realizing the fluorescence imaging of the cancer cells or tissues with high expression of CD47 (Ma et al., 2017). Photoacoustic imaging tomography (PAT) is a new biomedical imaging modality. Materials with light absorption capacity can produce sound waves. The principle of photoacoustic imaging is based on the photoacoustic effect of light-absorbers. Compared with the traditional *in vivo* optical imaging, photoacoustic imaging significantly enhanced imaging depths and spatial resolution (Wang and Hu, 2012). Due to the strong absorbance in the near-infrared region, 2D TMDCs could realize effective photoacoustic signal conversion, and be used as a light absorbent for photoacoustic imaging. For example, after being intravenously injected with WS_2_-PEG nanosheets, the mice bearing 4T1-tumors were imaged under a PAT imaging system with a 700 nm laser as excitation source. Remarkably enhanced PA signals were observed in the area of tumor, which indicating more tumor accumulation of WS_2_-PEG nanosheets (Cheng et al., 2014). In addition, TiS_2_ nanosheets with appropriate surface modification could also be effectively used *in vivo* photoacoustic imaging, and enhanced the efficiency of subsequent photothermal therapy (Qian et al., 2015). Since the mass extinction coefficient of graphene is far less than that of 2D TMDCs, and only materials with strong light absorption ability can generate sound waves, compared with 2D TMDCs, graphene is not a better choice for photoacoustic imaging (Chou et al., 2013). As a widely used imaging technology, X-ray CT imaging has the advantages of high resolution and deep tissue penetration. The principle of X-ray CT imaging is to reduce the intensity of X-ray and improve the contrast imaging ability by the strong X-ray attenuation ability of contrast agent (Shilo et al., 2012). Multiple nanomaterials containing elements with high atomic number have good X-ray attenuation ability (Liu et al., 2012a; Liu Z. et al., 2012). Due to the high atomic number and the strong X-ray attenuation ability of transition metal, 2D TMDCs are excellent contrast agents for X-ray CT imaging. After intravenous injection, both chitosan modified MoS_2_ (Yin et al., 2014) and WS_2_-PEG nanosheets (Cheng et al., 2014) exhibited excellent X-ray CT imaging ability in mice. Compared with 2D TMDCs, the X-ray attenuation ability of carbon element with smaller atomic number is weaker, so graphene is difficult to be a better contrast agent for X-ray CT imaging. In previous studies, graphene must be combined with other nanomaterials to form a composite material, which could be used in X-ray CT imaging (Zhang Y. et al., 2017). For example, after depositing graphene oxide on the surface of microcapsule containing gold nanoparticles, this microcapsule could serve as effective contrast agent to enhance X-ray CT imaging *in vitro* and *in vivo* (Jin et al., 2013).

### Biosensing

**FIGURE 8:**
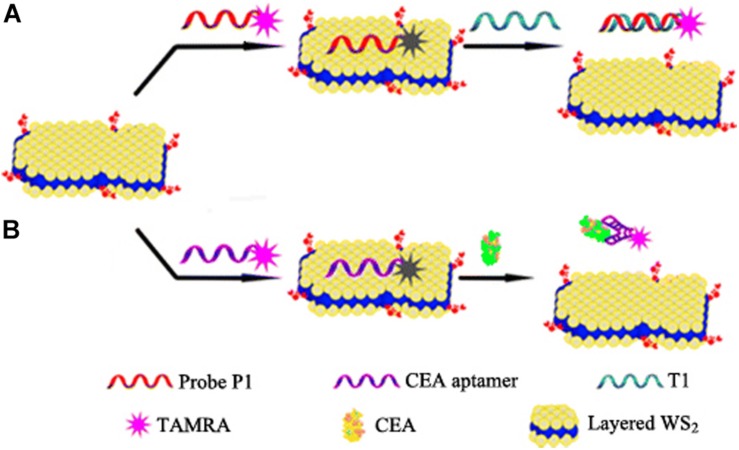
Schematic illustration of fluorescence sensing of nucleic acid and protein with layered WS_2_ nanosheet as the quencher. Two biosensors utilizing two different target recognition models, i.e., a nucleic acid hybridization model **(A)** and a protein–aptamer reaction model **(B)**, respectively. Reproduced with permission from Yuan et al. (2014).

As a novel biosensing platform, 2D TMDCs have two significant advantages. On the one hand, the super high surface area of 2D planar structure can fix a large number of sensing molecules to reach a very low detection limit. On the other hand, 2D TMDCs can excite the fluorescence group to the conduction band of 2D nanosheets through the photoinduced electron transfer effect, realizing the fluorescence quenching effect. In sum, 2D TMDCs are expected to be more advantageous biosensing platforms for detection of DNA (Figure 8; Zhu et al., 2013; Farimani et al., 2014; Yuan et al., 2014; Dou et al., 2017; Lu et al., 2017) and other small molecules (Xi et al., 2014). The fluorescent molecules-labeled single-strand DNA can be adsorbed on the surface of the nanosheets through the van der Waals interaction between the nucleobases and the basal plane of MoS_2_ nanosheets, which makes the single-strand DNA fluorescence quenching. When single-strand DNA was hybridized with its complementary DNA to form double-strand DNA, the interaction between the single-strand DNA and MoS_2_ nanosheets were greatly weakened. The single-strand DNA separated from the MoS_2_ nanosheets and the fluorescence quenched by MoS_2_ nanosheets can be retained, successfully detecting DNA molecules by MoS_2_ nanosheets. The detection limit of the biosensor based on MoS_2_ nanosheets for DNA was 500 PM and 5 μm for adenosine. This homogeneous process can be completed within a few minutes, showing a great application prospect of 2D TMDCs in biosensing and molecular diagnosis (Zhu et al., 2013).

## Biosafety Evaluation of 2D TMDCs

Two-dimensional TMDCs have emerged as promising materials for catalysis (Lukowski et al., 2013), photoelectronic devices (Yu P. et al., 2017; Si et al., 2018), energy storage (Wang T. et al., 2017), biosensing (Zhu et al., 2013), photodynamic therapy (Liu et al., 2014a; Yong et al., 2014), drug and gene delivery (Liu et al., 2014b; Kim et al., 2016; Ariyasu et al., 2017; Ma et al., 2017), due to various unique optical, electronic, mechanical, and chemical properties. The increasing applications of 2D TMDCs have increased their environmental accumulation and the possibilities of human exposures. Two-dimensional TMDCs as airborne particles, which are generated during production, usage, transportation and disposal of 2D TMDC-based products, will reach human respiratory system. In addition, the applications of 2D TMDCs in biomedical field enable their direct injection into circulation and accumulation in human organs and tissues. As foreign materials, 2D TMDCs certainly will disturb the normal physiological state. The biosafety evaluation of 2D TMDCs is of great significance to human health. The investigations on the biosafety of 2D TMDCs is still in its infancy. In this part, we make a summary on the basis of present research.

Cell death is the most serious consequence caused by nanoparticles. Compared to other disturbances of cell function caused by 2D TMDCs, research on cell viability affected by 2D TMDCs started earlier. For 2D TMDCs without surface modification, cell viability measurement showed a big difference between MoS_2_, WS_2_, and WSe_2_ nanosheets. The degree of cytotoxicity can be ranked in the order of WS_2_ < MoS_2_ < WSe_2_ (Teo et al., 2014). A possible cause of this situation is that the chalcogens are mainly located on exterior of each 2D TMDCs layer, allowing more interaction with cells compared with transition metal. Likewise to the case between H_2_Se and H_2_S, H_2_Se is much more toxic than H_2_S (Shamberger, 1981), selenium might be more hazardous than sulfur in 2D TMDCs, thus explaining above results. Compared with the 2D graphene, the cytotoxicity of various 2D TMDCs is lower than that of graphene (Teo et al., 2014). In theoretical simulation, graphene cut and inserted into cell membranes and extracted phospholipids, causing physical damage (Shi et al., 2016). However, when MoS_2_ nanoparticles interacted with cells, they sank into the phospholipid bilayer of cell membrane in a way parallel to the cell membrane, hardly causing physical damage to the cell membrane (Zhou et al., 2019). We speculate that the sharp edge of graphene may be the main reason for its high cytotoxicity. However, more deeply research is needed to determine if this speculation is valid. Under the same thickness and size, chemical composition was probably the major factor contributing to the variation in cytotoxicity of 2D TMDCs.

In addition to chemical composition, surface modification also produced an effect on the *in vitro* toxicity of 2D TMDCs. After incubation with MoS_2_ or chitosan-functionalized MoS_2_, the viabilities of two kinds of human cells showed that chitosan-functionalized MoS_2_ was more biocompatible than unmodified MoS_2_ nanosheets, indicating the significance of chitosan functionalization in decreasing cytotoxicity of 2D TMDCs (Yin et al., 2014). For WS_2_ nanosheets, another typical 2D TMDCs, surface modification also greatly reduced the toxicity *in vitro*. After incubation with three kinds of human cells, respectively, PEGylated WS_2_ exhibited negligible cytotoxicity. In contrast, without PEGylated WS_2_ nanosheets were obviously toxic to human cells. After treated with unmodified WS_2_ nanosheets, the cell survival rates of three kinds of human cells were less than 50%. This clearly suggested that the cytotoxicity of WS_2_ nanosheets was also closely associated with surface modification, which was consistent with MoS_2_ nanosheets (Cheng et al., 2014). In addition, several other surface-modified 2D TMDCs exhibited very low cytotoxicity even at higher concentrations in different human cells (Yong et al., 2014; Wang et al., 2015b; Yu et al., 2015; Ariyasu et al., 2017). Based on the existing limited research, it is probably that the surface modification could significantly decreased the cytotoxicity of 2D TMDCs. The specific reasons of this phenomenon need further study.

As an important physicochemical property of 2D nanomaterials, thickness played critical role in cell death induced by 2D TMDCs. After obtaining three MoS_2_ nanosheets of different thicknesses by different exfoliation methods, the *in vitro* toxicity of these three nanosheets to human lung cells was compared. Tert-butyllithium and n-butyllithium exfoliated MoS_2_ nanosheets were more cytotoxic than methyllithium exfoliated MoS_2_. Tert-butyllithium and n-butyllithium provided more efficient exfoliation than methyllithium. In other words, thickness was a factor influencing the cytotoxicity of MoS_2_ nanosheets. The smaller the thickness of the MoS_2_ nanosheets, the stronger their cytotoxic influence (Chng et al., 2014). The increase in active edge sites and surface area might contribute to the increased cytotoxicity caused by thickness reduction.

**FIGURE 9:**
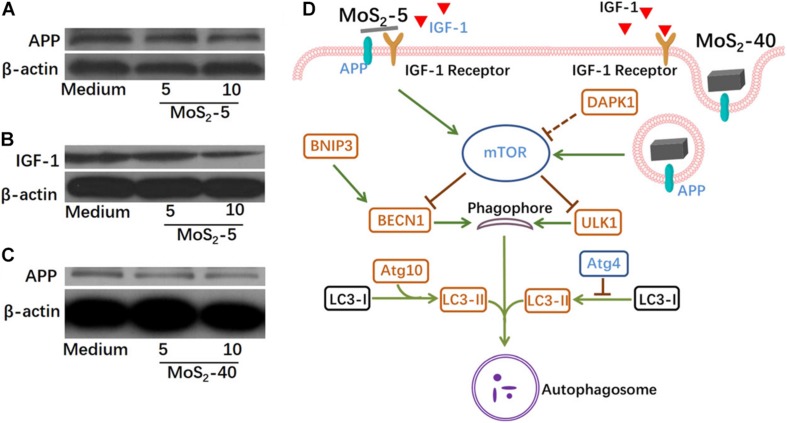
Probable interactions between MoS_2_ nanosheets and cell surface proteins and the possible perturbations of autophagy-related cell signaling. Immunoblot assay indicated that MoS_2_-5 inhibited APP **(A)**, IGF-1 **(B)**, and MoS_2_-40 inhibited APP **(C)**. **(D)** Signaling scheme showing the current understanding of the probable mechanisms for MoS_2_-5- or MoS_2_-40-induced autophagy. Reproduced with permission from Zhou et al. (2019).

However, the interaction between 2D TMDCs and cells certainly will disturb cell homeostasis. Further biosafety evaluation indicated that 2D TMDCs affected cell behaviors, such as cell proliferation, differentiation (Zou et al., 2017), cell adhesion, spreading (Suhito et al., 2017), oxidative stress (Yang et al., 2014), cell metabolism (Yu Y. et al., 2017), and cell autophagy (Zhou and Yan, 2019; Zhou et al., 2019). Single-layer MoS_2_ nanosheets promoted proliferation and accelerated myogenic differentiation in human embryonic lung fibroblasts (HELFs). The immunoblot assay and immunofluorescence analysis indicated the Akt-mTOR-p70S6K signaling pathway played a critical role in the induction of cellular proliferation and differentiation by single-layer MoS_2_ in HELFs (Zou et al., 2017). Based on the observation of cell morphology, human mesenchymal stem cells on MoS_2_ film exhibited more active filopodial interaction with substrate compared to the control group, indicating that MoS_2_ highly enhanced cell adhesion and spreading (Suhito et al., 2017). In human hepatoma HepG2 cells, MoS_2_ nanosheets induced mitochondrial depolarization and elevated reactive oxygen species level (Liu et al., 2017). High levels of oxidative stress induced by single-layer MoS_2_ nanosheets caused disturbance to Akt related signaling pathway (Zou et al., 2017) and enhanced antibacterial activity (Yang et al., 2014). In addition, chitosan functionalized MoS_2_ nanosheets activated TGF-β/Smad pathway and disturbed cellular metabolic process through interacting with EGFR on human dermal fibroblasts cells surface (Yu Y. et al., 2017). WS_2_ nanosheets induced obvious alterations in metabolic pathways and metabolites and the phase composition made a difference on metabolomics in algae (Yuan et al., 2018). Both the cell surface adhesion of thinner MoS_2_ nanosheets and cell internalization of thicker MoS_2_ nanosheets activated mTOR-dependent autophagy signaling pathway through perturbing cell surface protein amyloid precursor proteins (Figure 9; Zhou et al., 2019).

This phenomenon of 2D TMDCs is similar to that of graphene, which can also cause different degrees of cell function disturbance in a variety of cells, including ROS, proliferation and apoptosis (Pelin et al., 2018; Alsaedi et al., 2019; Liu X. et al., 2019). For example, graphene induced autophagy by activating NFκB and TLR signaling pathways in THP-1 cells and RAW 264.7 cells, respectively. Although both graphene and 2D TMDCs induced cell autophagy, the specific upstream initiation approaches were different (Chen et al., 2012; Qin et al., 2015). We speculate that the size, thickness, surface modification, and element composition may contribute to the differences between graphene and 2D TMDC in the disturbance of cell function. Preliminary evaluation has demonstrated that in the case of low cytotoxicity 2D TMDCs caused disturbances to multiple basic cell behaviors. However, the existing research disregarded the effects of cell line types and physicochemical properties of nanosheets on the cell perturbation induced by 2D TMDCs. More systematic biological and biosafety evaluation of 2D TMDCs are urgently required to further determine their potential risks and ensure their safety application.

## Conclusion and Pespective

This review summarized the synthesis methods, modification methods, important biomedical applications, and biosafety evaluation of 2D TMDCs. Driven by the wide application prospect of 2D TMDCs, remarkable progress has been made in their synthesis methods in recent years. At the same time, in order to obtain 2D TMDCs with various properties and realize their application in multiple fields, more and more attention has been paid on the surface modification of 2D TMDCs. The maturity of synthesis and modification methods promoted the wide application of 2D TMDCs in many fields, especially in biomedical field, increasing the opportunities of human exposure. As we all know, once entering human body, 2D TMDCs may interact with biological system and disturb homeostasis of physiological system. 2D TMDCs, as a kind of nanomaterial, can also pose a threat to human health. Therefore, it is necessary to evaluate the biosafety of 2D TMDCs. As 2D graphene analogs, most of studies on 2D TMDCs have been done just in the past few years, so the research in this field is still in its infancy. To further facilitate advances of this field, there are still several critical issues to be solved.

From the perspective of nanomaterials, the synthesis of 2D TMDCs lacks the standard method of controllability. It is still a big challenge to synthesize 2D TMDCs with desirable size and thickness. The size and thickness of 2D TMDCs obtained by the existing synthesis method are in a distribution range. Therefore, it is necessary to develop new synthetic methodologies of 2D TMDCs with desirable structural and compositional parameters. In addition, the investigations on the surface modification of 2D TMDCs have just been carried out. In order to realize the surface diversity modification of 2D TMDCs, it is essential to clarify other surface modification methods to expand the application space of 2D TMDCs.

From the biosafety evaluation point of view, a large number of studies have shown that the disturbance of the physiological system caused by nanomaterials is closely related to the properties of nanomaterials. As a new kind of 2D nanomaterials, 2D TMDCs is very different from other nanomaterials. The research on the disturbance of 2D TMDCs to physiological system is still in its infancy. It is still unknown how the physicochemical properties, such as element composition, size, surface charge, and hydrophobicity, will affect the biological systems disturbance caused by 2D TMDCs. Hence it is necessary to systematically study how the various physiochemical properties affect the interaction between 2D TMDCs and physiological system and the specific molecular mechanism. On the other hand, the ultimate goal of the biosafety evaluation of 2D TMDCs is to reveal the potential risks of 2D TMDCs to human health. The conclusions obtained *in vitro* need to be further verified by *in vivo* experiments. Due to the complexity of the internal environment, the dosage, administration time, administration mode and model animals should be fully considered in the specific study. Finally, the possible physiological disturbance caused by 2D TMDCs and the specific effects of physicochemical properties are clarified through the systematic study on the internal level.

## Author Contributions

XZ designed this work of review, performed the literature search of the databases, and wrote the manuscript. HS and XB revised the manuscript. All authors approved the manuscript for publication.

## Conflict of Interest

The authors declare that the research was conducted in the absence of any commercial or financial relationships that could be construed as a potential conflict of interest.
